# Development of a Novel in Silico Model to Investigate the Influence of Radial Clearance on the Acetabular Cup Contact Pressure in Hip Implants

**DOI:** 10.3390/ma11081282

**Published:** 2018-07-25

**Authors:** Saverio Affatato, Massimiliano Merola, Alessandro Ruggiero

**Affiliations:** 1 Laboratorio di Tecnologia Medica, IRCCS—Istituto Ortopedico Rizzoli, 40136 Bologna, Italy; massimiliano.merola@ior.it; 2 Department of Industrial Engineering, University of Salerno, 84084 Salerno, Italy; ruggiero@unisa.it

**Keywords:** finite element analysis, total hip arthroplasty, UHMWPE, musculoskeletal multibody model, tribology

## Abstract

A hip joint replacement is considered one of the most successful orthopedic surgical procedures although it involves challenges that must be overcome. The patient group undergoing total hip arthroplasty now includes younger and more active patients who require a broad range of motion and a longer service lifetime of the implant. The current replacement joint results are not fully satisfactory for these patients’ demands. As particle release is one of the main issues, pre-clinical experimental wear testing of total hip replacement components is an invaluable tool for evaluating new implant designs and materials. The aim of the study was to investigate the cup tensional state by varying the clearance between head and cup. For doing this we use a novel hard-on-soft finite element model with kinematic and dynamic conditions calculated from a musculoskeletal multibody model during the gait. Four different usual radial clearances were considered, ranging from 0 to 0.5 mm. The results showed that radial clearance plays a key role in acetabular cup stress-strain during the gait, showing from the 0 value to the highest, 0.5, a difference of 44% and 35% in terms of maximum pressure and deformation, respectively. Moreover, the presented model could be usefully exploited for complete elastohydrodynamic synovial lubrication modelling of the joint, with the aim of moving towards an increasingly realistic total hip arthroplasty in silico wear assessment accounting for differences in radial clearances.

## 1. Introduction

Total hip replacement (THR) is the most successful application of biomaterials in the short term in order to alleviate pain, restore joint architecture, and increase functional mobility in diseased traumatized joints [1]. A major limiting factor to the service life of THRs remains the wear of the polyethylene acetabular cup. Preclinical endurance testing has become a standard procedure to predict the mechanical performance of new devices during implant development. Wear tests are performed on materials and designs used in prosthetic implants [1,2,3] to obtain quality control and acquire further knowledge on the tribological behavior in joint prostheses. To gain realistic results, a wear test should replicate the in vivo working conditions of the artificial implants [4]. However, it is well known that wear tests have a long duration and are expensive [1,5,6]. The simulation is run for several million cycles; assuming that one million cycles correspond to one year in vivo [7,8,9]. The running-in period encompasses approximately the first half a million cycles [10]; therefore, the steady-state wear is assessed by measuring the wear when the running-in period is over. Wear is measured either gravimetrically, measuring the weight loss of the component, or through a direct analysis of the volume that has been removed, optically or by coordinate-measuring [4,11,12,13].

Although in vitro wear evaluation of new medical devices is standardized [14], wear tests are not flexible enough. The prediction of wear in hip replacements has been a subject of intense study in recent years [15]. In silico investigation, where algorithms could be developed to model a biomedical process, is a logical extension of controlled in vitro experimentation. It is the natural result of the explosive increase in computing power available to scientists; thus, numerical models could be used to predict results of a wear test with less time and cost. Obviously, the in silico analysis integrates but does not replace the experimental tools.

Finite element analysis (FEA) has been widely used in many areas of biomechanics and biomechanical engineering to study parameters and boundary conditions, which are not accessible experimentally [15,16]. The numerical modeling tool of FEA has been widely applied to analyze the behavior of articular cartilage, joints, and bone structures under compressive and tensile stresses [17,18,19,20,21]. It was also used to improve the design of hip joint prostheses and to minimize experimental tests [22]. Structural applications include the design and development of joint prostheses and fracture fixation devices [23]. To make computational wear simulation meaningful, better wear models are needed, and those can only be built on experimental data that call for systematic experiments in which the relationships between the wear and the material properties, movements, lubrication, and loading are investigated.

FEA is a useful way to investigate the effects of changing parameters such as load, velocity, contact geometry, material properties, etc. This technique was introduced to orthopedic biomechanics in 1972 to evaluate stresses in a human bone [24]. Since then, it has been utilized with increasing frequency in the biomedical engineering context. FEA uses algorithms in which a domain is realized by a number of sub-domains referred to as elements [25,26]. The behavior of each element is readily defined and understood by numerical equations, which allow the study of complex behaviors of the entire body.

Many researchers use FEA to investigate the mechanical and tribological behavior of the most widespread hip implants. Maxian et al. [27] developed an adaptive re-meshing model to study the wear evolution on a long-term regime (20 years of follow up). He found that sliding distance was mainly responsible for the volumetric wear more than polyethylene thickness. Hu and collaborators [28] developed, in 2001, a fully thermo-mechanical coupled finite element model of a total hip prosthesis. The model simulating the wear test in a hip simulator was used to evaluate the transient contact stresses and to predict the rise of temperature due to the friction for different applied loads, sliding speeds, and frictional coefficients. Gao and co-workers [15] developed a wear model, on metal-on-metal hip replacements, which considered lubrication for the first time via a transient elastohydrodynamic lubrication regime. Sfantos et al. [29], on the other hand, performed a parametric study of the wear in total hip arthroplasty by using a boundary elements method.

**Figure 1:**
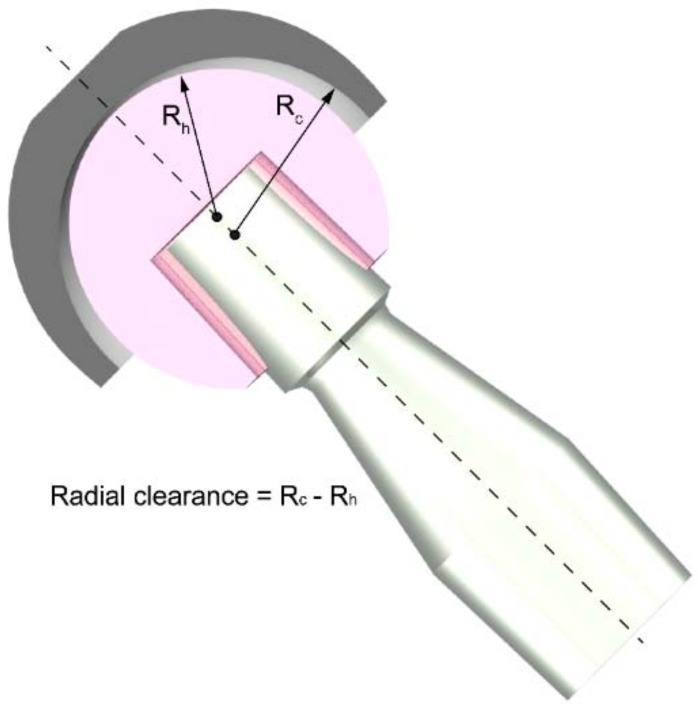
Schematization of the radial clearance between the inner surface of the acetabular cup (grey component in the picture) and the outer surface of the femoral head (pink component in the picture).

The aim of this study was to develop a flexible finite element model, starting from the dynamic data retrieved by means of a multibody musculoskeletal model of the lower limb. In particular, in a previous work Ruggiero et al. [30] used this multibody model to estimate the loads and kinematic forces acting on the hip joint during walking and modelling with these inputs the deformation of an acetabular cup. Our study reported here focused on the influence that the radial clearance, namely the difference between the inner radius of the cup (R_c_) and the radius of the femoral head (R_h_)—see Figure 1—has on the pressure and deformation of the acetabular cup in a hard-on-soft bearing.

## 2. Materials and Methods

### 2.1. Finite Element Analysis (FEA) Model

Workbench Ansys^®^ Software (v. 18.1, ANSYS Inc., Canonsburg, PA, USA) was used for the realization of the finite elements model. The model considered the hard-on-soft bearing configuration, typical of the most widespread hip implants [31]. To solve the contact problem, the solution algorithm must establish a relationship between the interacting surfaces, preventing the interpenetration. The software enforces contact compatibility through a penalty-based formulation that in this study is the augmented Lagrange, with a program-controlled penetration tolerance. With this formulation, a direct proportionality between the applied force and the penetration, where the coefficient is a contact stiffness, is established. Furthermore, the force is augmented by an extra term, making this method less sensitive to the magnitude of the contact stiffness. An asymmetric behavior was chosen for the contact, where the target body was the head and the contact body the cup, as all the resulting data belong to the contact side.

The simulations were performed comparing the effect of different clearances ranging from zero to 0.5 mm, namely 0, 0.05, 0.25 and 0.5 mm. The comparisons were in terms of magnitude and distribution of the contact pressure and of the deformation at the interface. As the sliding surfaces play a key role in the tribological performance of hip implants, the presence of the frictional force was considered. The coefficient of friction was obtained from experimental activities [32,33], considering a dry contact, and corresponded to 0.13. Even though the surface roughness could influence the friction and wear of a joint [34,35], the ease of computation and the very fine roughness—typical of actual hip replacements—led to a discharge of this aspect in the study. Contact area, pressure, and deformation were computed as a function of the angular displacements and dynamic loading calculated through a musculoskeletal model. Force components and orientations will be defined with respect to a pelvic reference frame that coincides with the true anatomic superior, anterior, and lateral directions.

Ultra-high-molecular-weight polyethylene (UHMWPE) type GUR 1050, was chosen for the acetabular cup whereas an infinitely rigid body was chosen as the femoral head, which is commonly realized in metal or ceramic. This choice is justified by the assumption that the site of wear interest is almost exclusively the soft body of the joint. Table 1 presents the main parameters of the acetabular cup material [36]. The presence of the pelvic bone has been neglected, since it has little influence on contact pressure [37]. The femoral head had a diameter of 28 mm, whereas the acetabular cup had a thickness of 5 mm. The cup was oriented with an anterior–posterior angle of 45°, and an inclination angle of 0°, these angles derived directly from the musculoskeletal model, from which forces and displacements were obtained. Therefore, it was not necessary to impose further inclination in the model.

**Figure 2:**
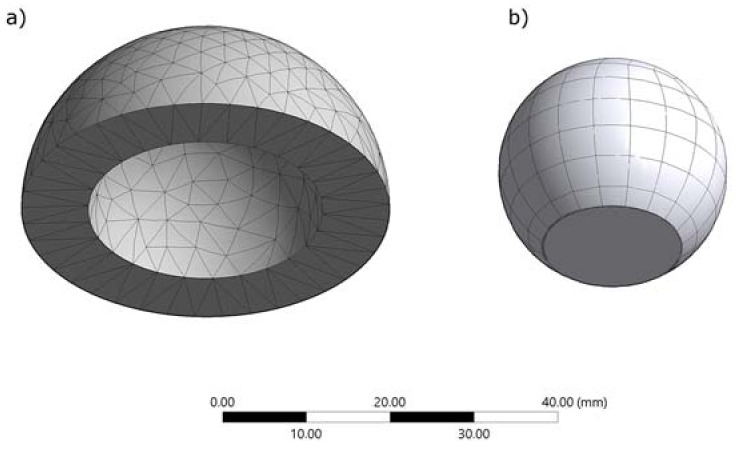
Meshing on (**a**) the acetabular cup and the (**b**) femoral head.

The finite element model is shown in Figure 2. A convergence study was performed to determine the optimum mesh aspect; contact pressure and cup deformation were used as performance metrics. For the cup the tetra patch conforming and tetra patch independent, the hex dominant quadrilateral/triangular and all quadrilateral, multizone hexagonal were compared; for the head, quadrilateral and triangular elements were compared. Regarding the head, quadratic elements were selected, for the cup a multizone hexagonal/prismatic hexagonal dominant meshing was chosen. The meshing of the entire model generated a total of 2012 elements and 3283 nodes, having 383 contact elements (Conta174 for the cup and Targe170 for the head). Elements of the mesh, having a minimum edge length of 53.7 mm, presented an average quality of 0.759 ± 0.152 and an average aspect ratio of 2.144.

**Figure 3:**
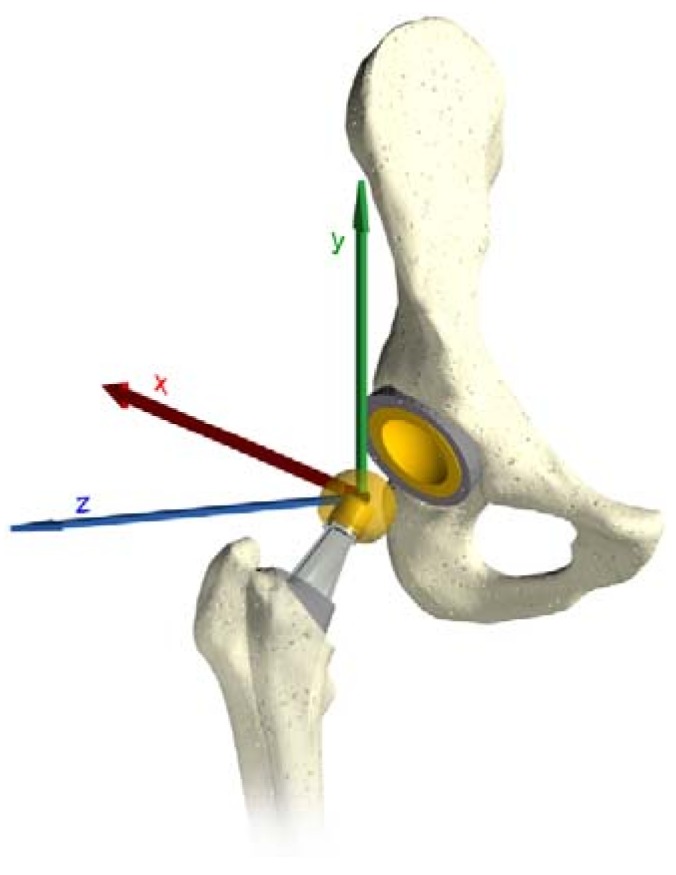
The three force directions.

The input data obtained by the multibody system are in a local coordinates system, which follows the movements of the femoral head. As the software requires a global coordinates system, a conversion was required by using the rotational matrix. The three rotations around the axes are the Flexion/Extension (*Z* axes), Abduction/Adduction (*X* axes) and Inward/Outward (*Y* axes) (see Figure 3).

### 2.2. Gait Cycles and Loads

In this study, we used musculoskeletal modeling software, to estimate loads acting on the hip joint during level walking. AnyBody Modelling System^TM^ (AnyBody Technology A/S, Aalborg, Denmark) is a musculoskeletal modeling and simulation software. It combines the principles of the inverse dynamics with different algorithms to define the muscle recruitment and analyze the loads acting on different joints of the human body [38]. To calculate the joint forces, the kinematic data and ground reaction force must be known and used as inputs. These data are collected using special motion capture tools in the gait analysis laboratories [39], which measure a subject gait by means of cameras that monitor markers on the person’s skin.

In inverse dynamics, the motion and the external loads on the body are known, and the aim is finding internal forces. Since not enough equilibrium equations are available to solve the problem, the evaluation of the muscle forces is made possible by the so-called redundancy problem. The working method of the AnyBody Modelling System is amply defined in [38], stating that there is the need to use an algorithm to find the activation of the muscles to reproduce the function of the central nervous system. The resolution of the multibody problem is already described in a previous work [30].

## 3. Results

**Figure 4:**
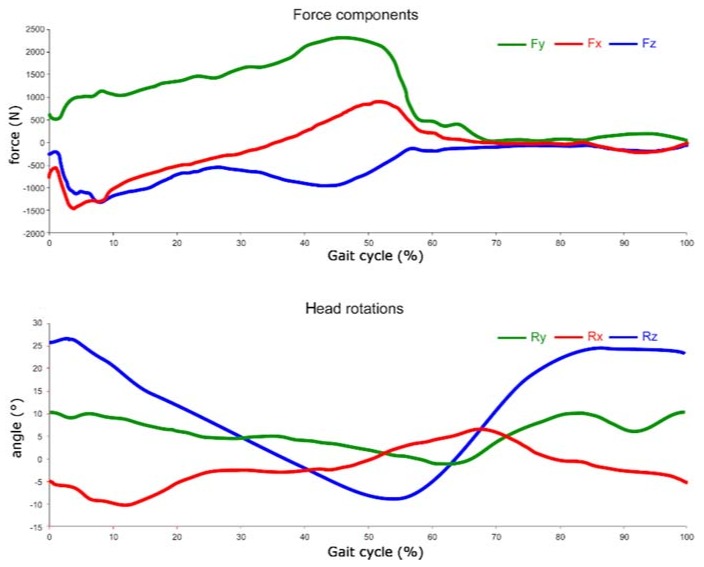
Forces and rotations obtained from multibody.

The force components and rotations, obtained from the multibody analysis, are shown in Figure 4, plotted along the gait cycle percentage.

**Figure 5:**
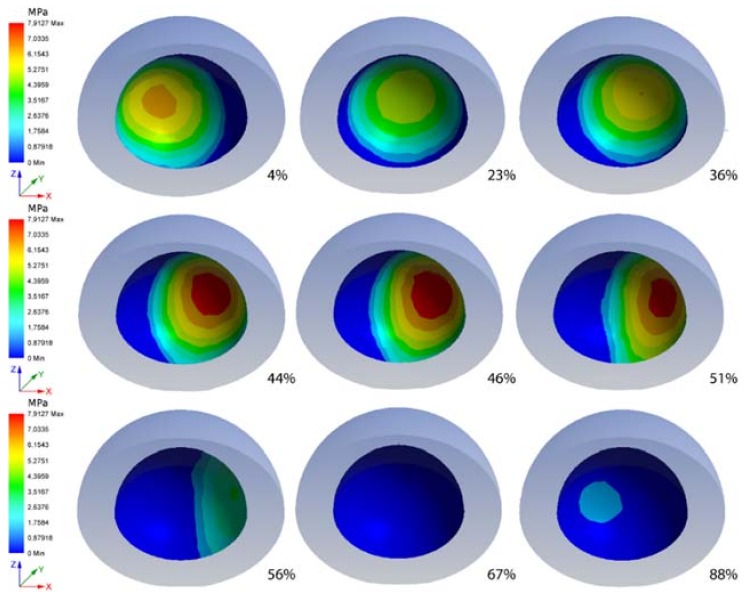
Pressure of the cup in several steps, as expresses in term of gait cycle percentage below each image.

In Figure 5, pressure distribution, on the sliding surface of the acetabular cup during different steps are shown. Along with the cycle progressing, it is possible to observe the distributions of pressure and its magnitude. The rotation of the head (suppressed in the images for a better understanding) varies with the position of the highest value of pressure. When the pressure reaches its maximum values along the cycle, its major magnitude is in the edge zone of the cup.

**Figure 6:**
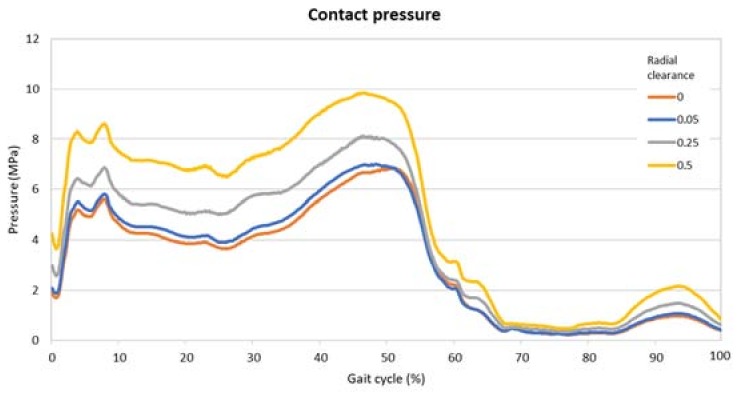
Maximum values of the pressure on the contact surface for different radial clearances.

**Figure 7:**
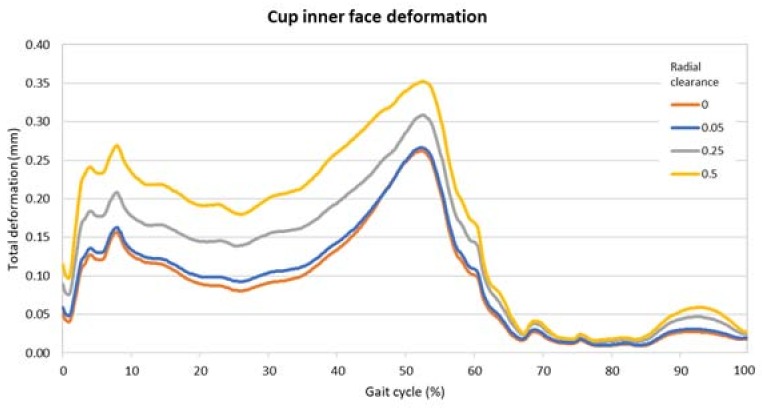
Maximum values of the deformation on the inner surface of the acetabular cup for different radial clearances.

In Figure 6 and Figure 7 are the comparisons of the radial clearance on the pressure and the deformation, expressed in terms of the maximum value found on the inner surface of the cup in each timeframe. From these images it is possible to highlight the divergences due to the clearance and the two peaks along the cycle. The presence of the two moments of high pressure and deformation already found in the previous analysis is clear.

Table 2 presents the highest values along the gait cycle found for pressure and deformation, comparing the radial clearances. These values were all located around the middle of the gait cycle, i.e., nearly 50%.

## 4. Discussion

The increasing number of tribology studies to analyze polyethylene wear in total hip arthroplasty confirms the need for improved understanding and new solutions to avoid the failure of an implant due to polyethylene wear. Wear tests are performed on biomedical materials to solve or reduce failures or malfunctions due to material loss. It is well known that pre-clinical validation is considered as an extension in the risk analysis task, and wear tests in prosthetic hip implants are necessary in order to acquire quality control and further knowledge about the tribological processes in joint prostheses [7]. The “gold standard” accepted preclinical method to evaluate the wear performance of a hip implant design in the laboratory is performed using a joint simulator that simulates the physiological loadings and movements of the patients; these machines give important outcomes about the expected behavior of a hip implant in clinical use [7,30]. FEA methodology has been used for many years as a numerical technique to solve engineering problems related to stress and strain analysis of static or dynamic loaded contacts. The technique incorporates the principles of Newtonian mechanics to model and animate the realistic behavior of such contacts [40,41]. In addition, experimental studies are currently used, but they are quite expensive and time-consuming. In addition, these studies can analyze only limited configurations and load conditions. Some authors [42,43,44,45] used different protocols than those recommended by the ISO standard for hip and knee joint simulation [14,46,47] considering highly demanding activities such as stair climbing, chair sitting, squats, etc. Unfortunately, all these experimental approaches are time-consuming and very expensive. For these reasons, the use of computational modeling is expanding also in this orthopedic field, but unfortunately, few published papers present validated wear models to use.

The aim of this study was to investigate the influence of the radial clearance on the pressure and deformation of an acetabular cup made of UHMWPE. The investigation based its input data on a musculoskeletal multibody model, solved for a walking cycle. The results showed the behavior of the inner face of the cup, which is subjected to a dynamic pressure, changing magnitude and orientation along the gait cycle. In detail, the side zones of the cup are subjected to an elevated pressure during the first and last touching moments of the feet on the ground, when there is the impact and the release. These instants are known as heel strike and toe off, and their occurrence corresponds to an elevated bending in flexion.

The values of pressure find their agreement with previous studies, like the one performed by Liu et al. [16], where the maximum pressure corresponded to 8 MPa. The study on wear performed by Mattei et al. [48] showed a contact pressure of 8.2 MPa in correspondence with the maximum load. In the work of Matsoukas et al. [49] the highest values were almost 8 MPa and 12 MPa for the von Mises pressure and the contact pressure, respectively. These values were found in correspondence with the second peak of the stance phase, plus the pressure distribution concentrates in the edge zone of the cup. Maxian et al. [50] found a similar distribution of pressure reaching a maximum value of 9 MPa. The small differences that we found can be attributed to the dynamic loading retrieved from the musculoskeletal model, which differ from the standardized loads used in the literature. It is believed that before improving and making a more complex algorithm to find wear in silico, taking also into account realistic lubrication models, there is a need to apply more flexible and reliable loads as used in this study.

The comparison across the different radial clearance values highlighted that the there is a sensible divergence in term of pressure and deformation. From the 0 value of radial clearance to the highest, 0.5, there is a difference of 44% and 35% in terms of maximum pressure and deformation, respectively. This increment can be attributed to the modification of the contact distribution. In fact, when two convex–concave bodies pressing against each other are in perfect compliance the load is distributed across a wider area than if the same two bodies differ in dimension. Having a highly conformal contact means a wide area where forces are distributed and therefore a smaller pressure amplitude. As a consequence of a less intense pressure, the maximum values of the deformation are also smaller.

There is a very small variation between the two close cases of no clearance and 0.05 mm. In detail, the difference of these conditions is limited to the 3% of the maximum pressure and 4% of the maximum deformation. This variation is so little that it can be discarded in a first approximation, but even such a small difference can be relevant when lubrication comes into play. Lubrication in fact is dependent on different factors such as the viscosity of the lubricant, the finishing of the surfaces, and the shape of them. Research has demonstrated that lubricated hip joints could be modelled as a ball-in-socket or ball-on-plane equivalent configuration in boundary, mixed or hydrodynamic/elastohydrodynamic lubrication regimes. In this configuration, small radial clearances may benefit from lubrication phenomena by increasing minimum film thickness. Moreover, the greater entraining velocity in correspondence with low clearances could favor the hydrodynamic lubricating fluid film with a consequential positive effects on wear [24,51,52,53,54].

Obviously, our study has a number of limitations. The first limitation is due to the fact that our model was not validated experimentally on an in vitro simulation but it was validated only by comparison with international literature. It is stressed, however, that our model is in agreement with other in silico models. The second limitation is that our model was developed for a diameter of 28 mm, further studies are necessary to find out if there are differences with other dimensions. The model considered a single orientation of the cup, which is believed to be valid as stated by other studies [22], but limits its application to the specific case. Different cup inclination angles can influence the contact zone, as reported in the work of Hua et al. [55]: By increasing the cup inclination “the microseparation distances required to generate edge loading decreased”. Other factors, such as additional effects of contact mechanics, or mechanics associated with three-body wear, temperature, and lubrication were not incorporated in this model and remain to be modelled.

## 5. Conclusions

The proposed finite element model allowed us to investigate the tribo-mechanical behavior of a hip implant subjected to dynamic loads derived from a multibody musculoskeletal model. The study focused on the differences found in pressure and deformation, in terms of distribution and amplitude, by varying the radial clearance in the range of 0–0.5 mm. The results highlighted the following main findings:

- along the walking cycle, the heel strike and the toe off are the instants of highest pressure, against these points the maximum pressure is located on the lateral side of the cup;
- radial clearance plays a substantial role on pressure and deformation magnitude across the gait cycle: the higher the clearance, the higher the pressure. Nevertheless, reducing the clearance to the smallest possible value is believed to affect the bearing lubrication;
- since radial clearance plays a key role in the lubricating phenomena, the proposed finite element model will be useful to exploit in the complete lubrication modelling of the joint toward a more and more realistic assessment of the in silico wear, accounting also for different radial clearances.

## Figures and Tables

**Table 1:** UHMWPE GUR 1050 properties.

Density	Young’s Modulus	Poisson’s Ratio	Bulk Modulus	Shear Modulus	Tensile Yield Strength	Tensile Ultimate Strength
(kg m^−3^)	(MPa)	(-)	(MPa)	(MPa)	(MPa)	(MPa)
930	690	0.46	1640	241	21	40

**Table 2:** Maximum values along the gait cycle of the pressure and deformation on the inner face of the cup.

Radial clearance	0	0.05	0.25	0.5
Maximum pressure (MPa)	6.82	7.00	8.12	9.84
Maximum deformation (mm)	0.26	0.27	0.31	0.35
